# Tumor Necrosis Factor Alpha Blocking Agents as Treatment for Ulcerative Colitis Intolerant or Refractory to Conventional Medical Therapy: A Meta-Analysis

**DOI:** 10.1371/journal.pone.0086692

**Published:** 2014-01-27

**Authors:** Ruxi Lv, Weiguang Qiao, Zhiyong Wu, Yinjun Wang, Shixue Dai, Qiang Liu, Xuebao Zheng

**Affiliations:** 1 School of Traditional Chinese Medicine, Southern Medical University, Guangzhou, People's Republic of China; 2 Research Institute of Traditional Chinese Medicine, Guangdong Medical College, Zhanjiang, Guangdong, People's Republic of China; 3 Department of Gastroenterology, Nanfang Hospital, Southern Medical University, Guangzhou, People's Republic of China; 4 Emergency Department of Nanfang Hospital, Southern Medical University, Guangzhou, People's Republic of China; CWRU/UH Digestive Health Institute, United States of America

## Abstract

**Background:**

Efficacy of tumor necrosis factor alpha (TNF-α) blockers for treatment of ulcerative colitis that is unresponsive to conventional therapy is unclear due to recent studies yielding conflicting results.

**Aim:**

To assess the efficacy and safety of anti-TNF-α agents for treatment of ulcerative colitis patients who were intolerant or refractory to conventional medical therapy.

**Methods:**

Pubmed, Embase, and the Cochrane database were searched. Analysis was performed on randomized controlled trials that assessed anti-TNF-α therapy on ulcerative colitis patients that had previously failed therapy with corticosteroids and/or immunosuppressants. The primary outcome focused on was the frequency of patients that achieved clinical remission. Further trial outcomes of interest included rates of remission without patient use of corticosteroids during the trial, extent of mucosal healing, and the number of cases that resulted in colectomy and serious side effects.

**Results:**

Eight trials from seven studies (n = 2122) met the inclusion criteria and were thus included during analysis. TNF-α blockers demonstrated clinical benefit as compared to placebo control as evidenced by an increased frequency of clinical remission (p<0.00001), steroid-free remission (p = 0.01), endoscopic remission (p<0.00001) and a decrease in frequency of colectomy (p = 0.03). No difference was found concerning serious side effects (p = 0.05). Three small trials (n = 57) comparing infliximab to corticosteroid treatment, showed no difference in frequency of clinical remission (p = 0.93), mucosal healing (p = 0.80), and requirement for a colectomy (p = 0.49). One trial compared infliximab to cyclosporine (n = 115), wherein no difference was found in terms of mucosal healing (p = 0.85), colectomy frequency (p = 0.60) and serious side effects (p = 0.23).

**Conclusion:**

TNF-α blockers are effective and safe therapies for the induction and maintenance of long-term remission and prevention of treatment by colectomy for patients with refractory ulcerative colitis where conventional treatment was previously ineffective. Furthermore, infliximab and cyclosporine were found to be comparable for treating acute severe steroid-refractory ulcerative colitis.

## Introduction

Ulcerative colitis (UC) is a chronic disease characterized by diffuse mucosal inflammation within the colon, often with alternating periods of exacerbation and remission. This disease has conventionally been treated with 5-aminosalicylic acid, corticosteroids and oral immunosuppressant (e.g. azathioprine, 6-mercaptopurine) with the goals of achieving clinical or mucosal remission, and/or eliminating long-term corticosteroid use [1]. However, these conventional therapies are in many instances ineffective or cannot be tolerated by the patients. This failure to pervasively treat UC patients is apparent in the frequency of colectomies performed; the cumulative probability of colectomy from the time of diagnosis is 13.1% at 5 years, 18.9% at 10 years, and 25.4% at 20 years [2]. This deficit in widespread, effective treatment of UC patients therefore warrants the development and study of alternative treatments.

One potential alternative therapy is inhibition of tumor necrosis factor alpha (TNF-α) as previous studies have established a correlation between increased production of TNF-α and UC pathophysiology [3]–[6].

Currently, the anti-TNF-α agents most commonly used for UC treatment are infliximab (IFX) and adalimumab (ADA). Intravenous and subcutaneous administration of IFX and ADA, respectively, has been shown by some studies to be effective for treating moderately to severely active UC [7]–[10]. However, other studies pertaining to IFX treatment have yielded conflicting results [11]. Another anti-TNF-α agents, golimumab, induces and maintains clinical remission in patients with moderate to severe UC as evidenced by two recent trials [12], [13]. The need for alternative UC therapies, as well as the range and conflicting reports found from studies on anti-TNF-α therapeutics, encouraged us to perform a meta-analysis to analyze the efficacy of these agents for UC patients who were intolerant or refractory to conventional medical therapy.

Several systematic reviews and meta-analyses of TNF-α blockers as treatment for UC have been published in recent years [14]–[17]_ENREF_10. However, these failed to fully take into account heterogeneity between the trials analyzed, including differences in the severity of UC in patients studied, drugs administered within the control group, and the point at which patient follow-up concluded. Moreover, the doses of the anti-TNF-α agent varied between different studies that had been included. As expected, these discrepancies skewed the results of the previous meta-analyses. Because of this need to account for inconsistencies within previous analyses, as well as include recent findings concerning anti-TNF-α treatment, we conducted a meta-analysis of TNF-α blockers as therapy for UC patients intolerant or refractory to conventional medical treatment. It would be very helpful for decision-making for patients with UC who do not respond well to conventional treatments if we could provide currently available evidence for or against anti-TNF-α therapeutics in UC. To reduce heterogeneity and enhance comparability between studies during our meta-analyses, trials wherein only a single infusion of anti-TNF-α was administered or patient follow-up concluded within 12 weeks post first treatment were excluded. Furthermore, sub-analyses were executed within our meta-analyses to account for whether the control group received placebos or active intervention.

## Methods

### Search strategy

The databases Pubmed, Cochrane Library and Embase were searched for studies published between 1991 and July 20, 2013 containing the terms “(infliximab or adalimumab or certolizumab or golimumab or tumor necrosis factor alpha) and (inflammatory bowel disease or ulcerative colitis) and (trial*).” Furthermore, the reference lists of any studies previously identified as having met the inclusion criteria were manually reviewed to find additional relevant publications.

### Study selection

The titles and abstracts of published studies were screened independently by two investigators to determine whether they fulfilled the following inclusion criteria: (i) the studies had to be randomized controlled trials (RCTs) comparing anti-TNF-α therapies (e.g. adalimumab, certolizumab, golimumab, or infliximab) with the administration of a placebo or other intervention, and published in the English language, (ii) the UC patients of any age included had to have UC resistant to conventional therapy of corticosteroids and/or immunosuppressive agents, or refractory to intravenous corticosteroids, and, (iii) the patients had to have been given TNF-α blockers at least twice and monitored for at least 12 weeks after the initial dose of TNF-α blocker or control drug. The primary outcome measured was frequency of clinical remission, which was defined by each of the primary studies. Secondary outcomes recorded were the frequency of long-term mucosal healing, steroid-free remission, colectomy and severe side effects. Furthermore, reviews, case reports and abstracts that lacked sufficient information to determine if the above parameters were met were excluded.

### Outcome assessment

Unless otherwise defined in the primary study, clinical remission was defined either as a total Mayo score≤2 with no individual subscore exceeding 1 points, mucosal healing was defined as an endoscopy subscore of 0 or 1. The decision to perform a colectomy was made on clinical grounds. Serious side effects were defined by each primary study.

### Data extraction

All data and inclusion decisions were performed independently by two investigators. When there was disagreement between the reviewers, the cases in question were discussed and a decision to include or exclude a study was made by reviewer consensus. The information collected from each study included the type of study, number of patients enrolled in the study, experimental and control therapies used, side effects observed, duration of patient follow-up, patient baseline demographics, patient medical and UC-related history, concomitant therapy received by the patient and the trial outcomes. For instances where a patient dropped out of the study or where data was missing, an intention-to-treat principle was applied and these cases were considered as treatment failure.

### Assessment of risk of bias

This data collection and assessment was performed independently by two investigators, wherein any disagreements were resolved by discussion. Risk of bias was assessed as described in the Cochrane handbook [18]: by recording the method of random sequence generation, the method of allocation concealment, whether blinding was implemented, whether incomplete outcome data was reported, whether an intention-to-treat analysis was conducted, and whether there was evidence of selective reporting of outcomes. The quality of the RCTs was assessed by the Jadad scoring system by two independent investigators [19].

### Statistical Analysis

The meta-analyses were performed by using relative risk (RR) for dichotomous outcomes. Pooled estimates were presented with 95% confidence intervals (CIs). Sub-analyses were chosen based on the type of control group within the study (placebo or active interventions). Heterogeneity between studies was quantified by calculating I^2^ where p<0.10 was determined significant. Where there was evidence of heterogeneity, a random-effects model was used for pooling. Otherwise, a fixed-effects model was used. Funnel plots were not conducted to investigate publication bias as there were not enough studies included in each comparison to produce a meaningful analysis. All statistical analyses were executed on RevMan 5.2 software. Results were analyzed according to the intention-to-treat principle.

## Results

### Literature retrieval

**Figure 1:**
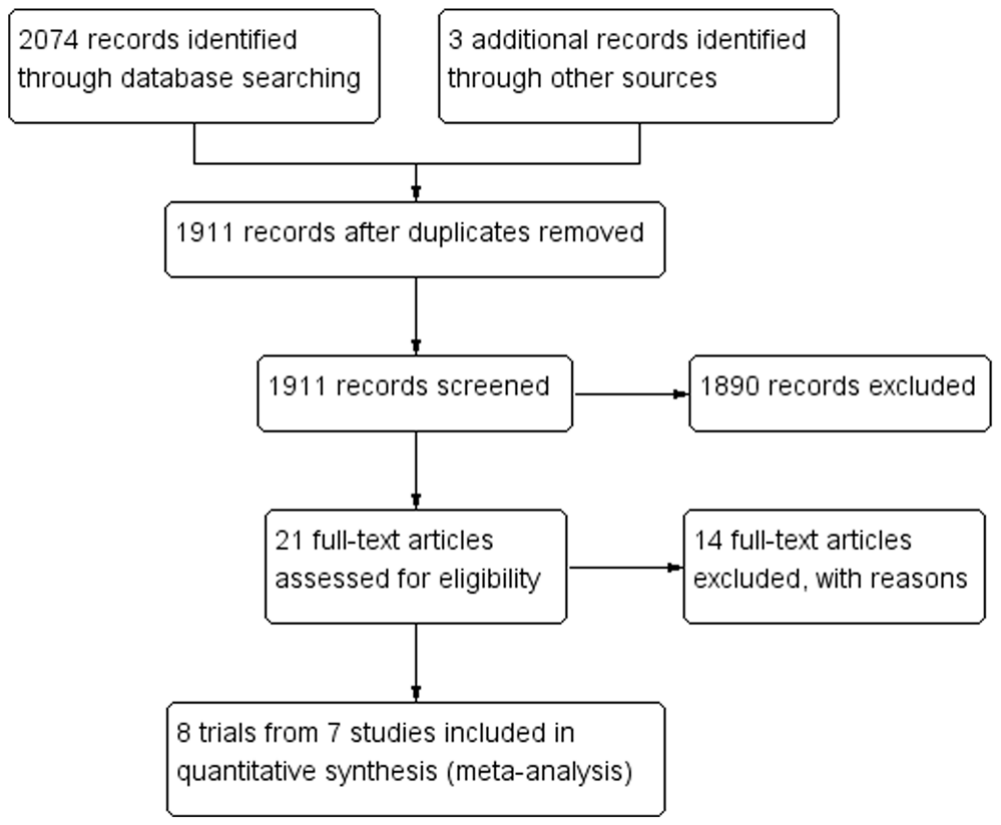
Study flow diagram.

The previously described search strategy identified 1911 citations, of which, 1890 were excluded after examination of the title and abstract (Figure 1). 21 articles reporting on the efficacy of anti-TNF-α therapies in UC were then further evaluated [8]–[13], [20]–[33]. 14 of these 21 articles were excluded: 4 due to use of only a single infusion of anti-TNF-α agents [20]–[23], 3 because the duration of patient follow-up lasted fewer than 12 weeks[10]–[12], 4 because the enrolled participants [13], [29], [30]or outcome(s) assessed[24] failed to meet the inclusion criteria, 1 because there was no placebo used [31], and 2 because the papers were published only as an abstract [32], [33].

The remaining 7 studies were used for meta-analysis [8], [9], [25]–[28], 1 study reported on 2 separate trials [8], bringing the total number of trials analyzed to 8. Of these trials, 4 compared infliximab or adalimumab treatment to placebo, 3 compared infliximab treatment to corticosteroid, and 1 compared infliximab to cyclosporine. The characteristics and trial design of the included studies were shown in Table 1 and Table 2, respectively.

**Table 1:** Baseline characteristics of included studies.

Study	Case (n)	Mean age (years)	Male (%)	Duration (years)	Co-therapy permitted	Type of study (Jadad score)
**Armuzi 2004**	20	36.3	-	5.15	NR	Open-label, RCT(2)
**Gavalas 2007**	24	47.8	58	4.64	AZA,Steriods,5-ASA	Controlled trial (3)
**Laharie 2012**	115	37.5	52.2	1.7	AZA, Antibiotics, nutritional; CS tapered.	Open-label, RCT (5)
**Ochsenkühn 2004**	13	37.4	46.2	5.5	Mesalazine, sulfasalazine, antibiotics, or anti-diarrheal drugs at stable doses	Double-blind, RCT (3)
**Rutgeerts 2005 ACT1**	364	41.9	74	6.8	CS alone or in combination with AZA or MP	Double-blind, RCT (6)
**Rutgeerts 2005 ACT2**	364	40.0	71.7	6.6	CS alone or in combination with AZA or MP and 5-ASA	Double-blind, RCT (6)
**Sandborn 2009**	728	41.0	60.0	6.7	CS and/or AZA or 6-MP and/or 5-ASA	Double-blind, RCT (6)
**Sandborn 2012**	294	40.4	59.5	8.3	CS and/or AZA or 6-MP; CS tapered	Double-blind, RCT (4)

**Note:** NR, Not reported; AZA, Azathioprine; 5-ASA, 5-aminosalicylates; CS, corticosteroids; MP, mercaptopurine; RCT, randomized controlled trail.

**Table 2:** Trial design of included studies.

Study	Participants(UC)	Intervention		Control	Follow-up	Outcome
**Armuzzi 2004**	Steroid-dependent	Infliximab		Methylprednisolone	9.8±1.1 months	Clinical remission; colectomy rate
**Gavalas 2007**	Steroid-dependent	Infliximab		Methylprednisolone	21months	Clinical remission
**Laharie 2012**	Not respond to intravenous steroid	Infliximab		Ciclosporin	98 days	Mucosal healing; colectomy rate; safety; serious adverse events.
**Ochsenkühn 2004**	Refractory to 5-aminosalicylates.	Infliximab		Prednisolone	13 weeks	Clinical remission; mucosal healing
**Rutgeerts 2005 ACT 1**	Not respond to conventional therapy	Infliximab		Placebo	54 weeks	Clinical remission; mucosal healing; steroid-free remission; serious adverse events.
**Rutgeerts 2005 ACT2**	Not respond to conventional therapy	Infliximab		Placebo	30-week	Clinical remission; mucosal healing; steroid-free remission; serious adverse events.
**Sandborn 2009**	Not respond to conventional therapy	Infliximab		Placebo	54 weeks	Colectomy rate; serious adverse events.
**Sandborn 2012**	Not respond to conventional therapy	Adalimumab	Placebo		54 weeks	Clinical remission; mucosal healing; steroid-free remission; serious adverse events.

**Note:** UC, Ulcerative colitis.

### Methodological quality of included studies

**Figure 2:**
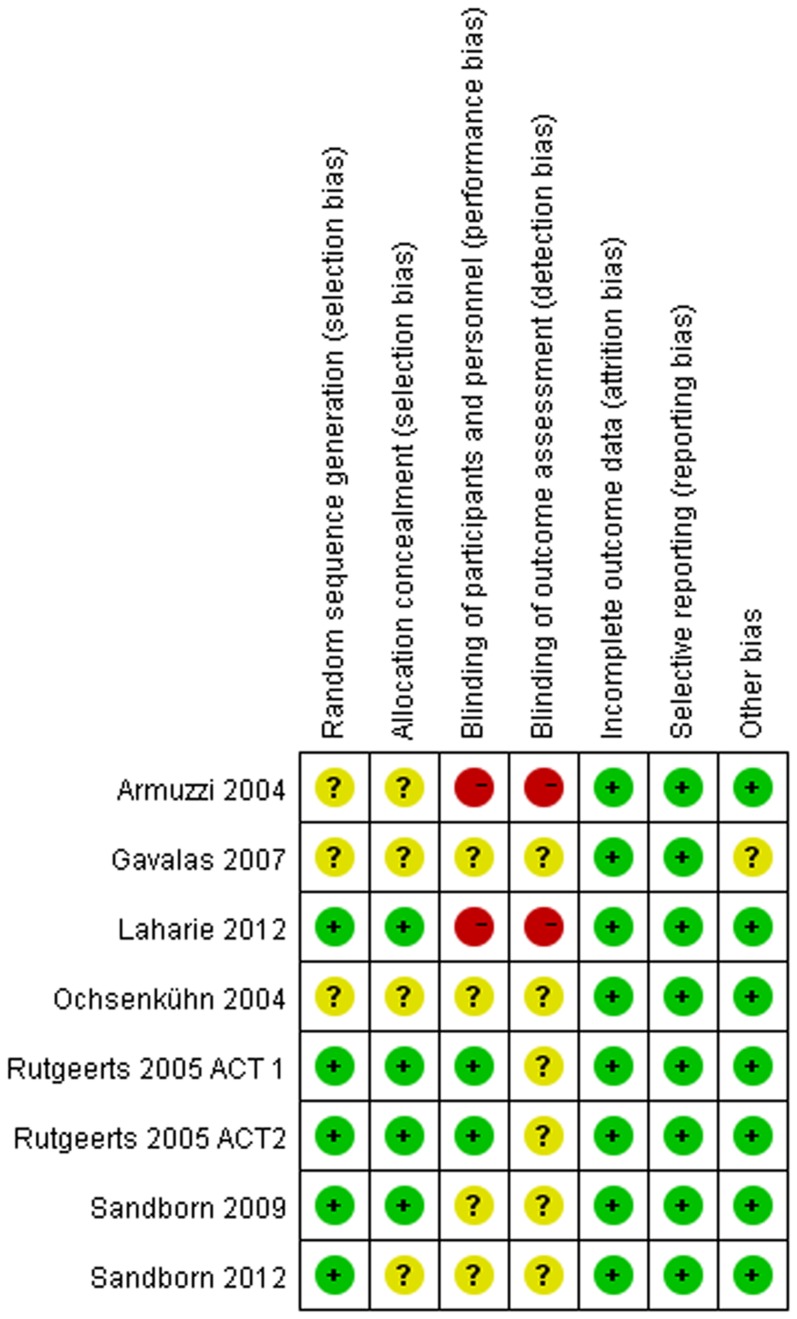
Risk of bias summary: review of authors' judgements about each risk of bias item for included studies.

**Figure 3:**
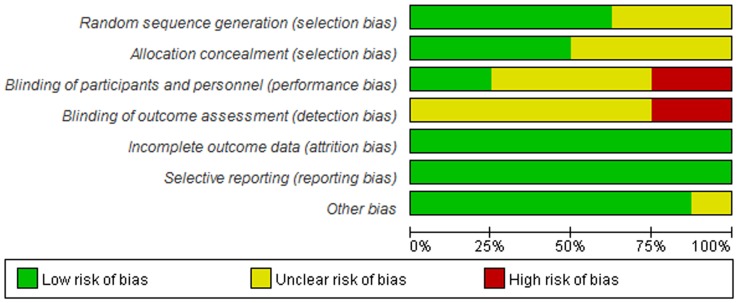
Risk of bias graph: review of authors' judgements about each risk of bias item presented as percentages across all included studies.

The assessment of the risk of bias was summarized in Figures 2 and Figure 3. Overall, the quality of the studies ranged from moderate to high (Jadad score≥3). Two studies were rated at high risk of bias due to lack of proper blinding controls [25], [27]. All data were analyzed in accordance with the intention-to-treat principle. Due to an insufficient number of studies to produce a meaningful analysis, funnel plots were not used to investigate publication bias.

### Data synthesis: Clinical remission

**Figure 4:**
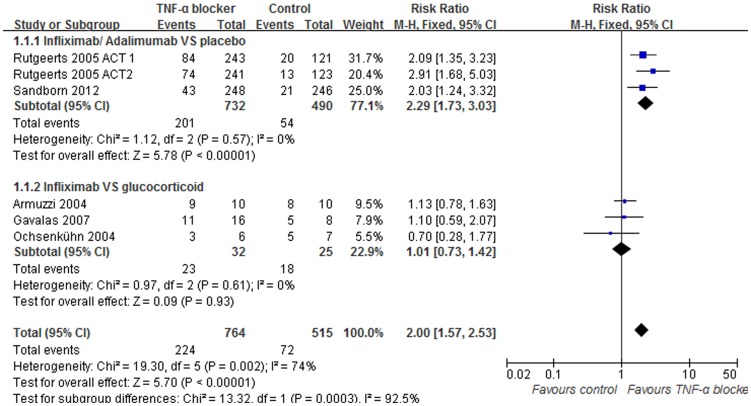
Pooled outcome for clinical remission in patients exposed to TNF-α blocker vs. controls.

The frequency of clinical remission of patients treated with TNF-α blockers was studied in 6 trials that consisted of 1279 patients. Of these 6 trials, 3 trials were controlled by administering a placebo. Patients were treated with infliximab in 2 of the trials and adalimumab in 1. No significant heterogeneity was detected between these trials (I^2^ = 0%, p = 0.57). A pooled analysis using fixed-effects models showed that the TNF-α blocker was significantly superior to placebo for maintenance of clinical remission (RR = 2.29; 95% [1.73, 3.03], Z = 5.78, p<0.00001, Figure 4). In 3 of the trials, infliximab treatment was compared with glucocorticoid. The control group within these trials consisted of patients given methylprednisolone in 2 of the trials and prednisolone in the other trial. There was no significant heterogeneity found among the trials (I^2^ = 0%, p = 0.61). Based on fixed-effects models, there was no significant difference in clinical remission rates between the anti-TNF-α agents and glucocorticoid treatment (RR = 1.01; 95% [0.73, 1.42], Z = 0.09, p = 0.93, Figure 4).

### Data synthesis: Mucosal healing

**Figure 5:**
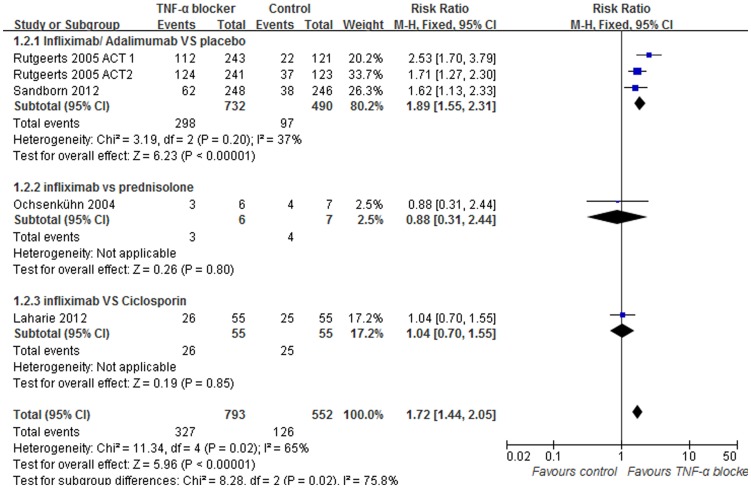
Pooled outcome for mucosal healing in patients exposed to TNF-α blocker vs. controls.

Mucosal healing was evaluated in 5 trials, consisting of 1345 patients, to determine TNF-α blocker treatment efficacy. Of these, 3 trials compared anti-TNF-α agents with a placebo control. Patients were given infliximab in 2 trials and adalimumab in the third trial. No heterogeneity was detected when comparing these 3 trials (I^2^ = 37%, p = 0.20). A pooled analysis using fixed-effects models showed the TNF-α blocker was significantly superior to placebo for healing of the mucosa (RR = 1.89; 95% [1.55, 2.31], p<0.00001, Figure 5). Only 1 trial included in our analysis compared infliximab with prednisolone. This trial found that infliximab and prednisolone are equally effective for sustaining mucosal healing in UC (RR = 0.88; 95% [0.31, 2.44], p = 0.80, Figure 5), although with the caveat of a small trial population. In another trial, patients within the control group were given cyclosporine, and it was concluded that infliximab is as effective as cyclosporine in sustaining mucosal healing in UC (RR = 1.04; 95% [0.70, 1.55], p = 0.85).

### Data synthesis: Steroid-free remission

**Figure 6:**
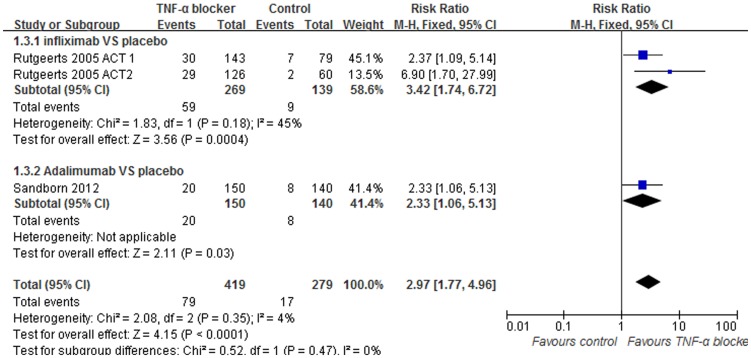
Pooled outcome for steroid-free remission in patients exposed to TNF-α blocker vs. controls.

Of the trials included in our analysis, 3, consisting of 698 patients, reported discontinued corticosteroid use and sustained steroid-free remission during their study. Of these, infliximab treatment efficacy was examined in 2 trials and adalimumab in 1 trial. No heterogeneity was detected when comparing the 3 trials (I^2^ = 4%, p = 0.35). A pooled analysis utilizing fixed-effects models was conducted. It was shown that the proportion of patients who achieved steroid-free remission was higher in groups that received the TNF-α blockers than in the placebo treated groups (RR = 2.97; 95% [1.77, 4.96], p<0.0001, Figure 6).

### Data synthesis: Colectomy rate

**Figure 7:**
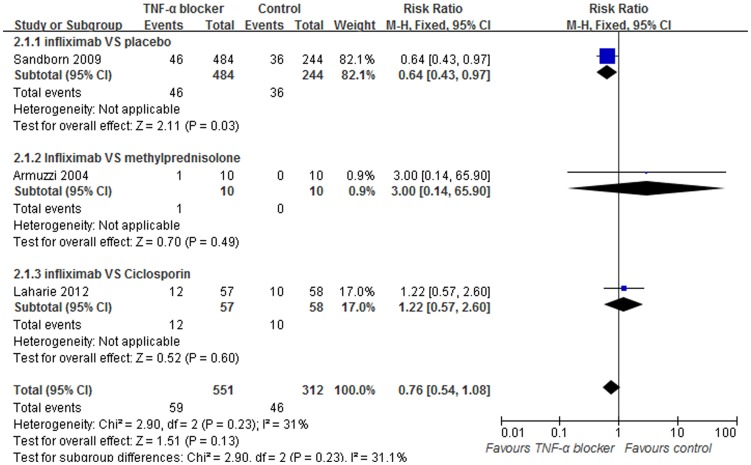
Pooled outcome for colectomy rate in patients exposed to TNF-α blocker vs. controls.

The rate of colectomy was only reported within 3 of the included trials, which evaluated a total of 863 patients. The data demonstrated that more patients in the placebo group (36/244) than in the infliximab group (46/484) had a colectomy, as shown in Figure 7. This difference in colectomy rate is statistically significant (RR = 0.64; 95% [0.43, 0.97], p = 0.03, Figure. 7), indicating the benefit of infliximab treatment. In another trial, methylprednisolone was showed that the colectomy rate was equivalent between those receiving infliximab and those receiving prednisolone (RR = 3.00; 95% [0.14, 65.90], p = 0.49, Figure 7). Finally, 1 trial administered cyclosporine within the control group. This trial found that infliximab is as effective as cyclosporine in preventing patient colectomy (RR = 1.22; 95% [0.57, 2.60], p = 0.60, Figure 7).

### Data synthesis: Serious side effects

**Figure 8:**
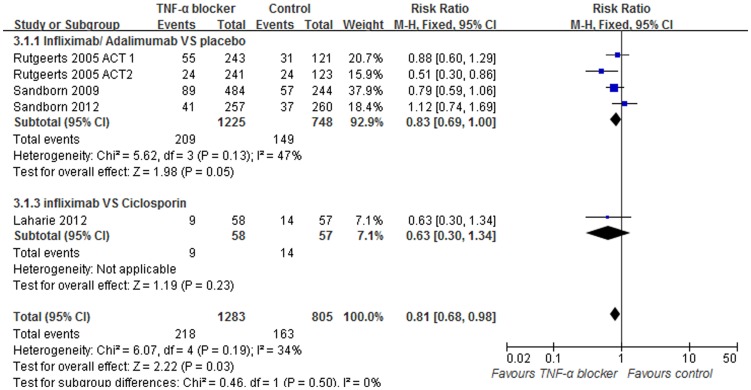
Pooled outcome for serious side effects in patients exposed to TNF-α blocker vs. controls.

Serious side effects were reported in 6 of the trials, consisting of 2088 patients. Within these trials, the frequency of serious side effects was 16.9% in the anti-TNF-α group, 20.0% in the placebo group and 24.7% in cyclosporine group. Of these, 4 trials administered a placebo as a control and 1 used cyclosporine. Significant heterogeneity was not detected when comparing these trials (I^2^ = 34%, p = 0.19). A pooled analysis using fixed effects models showed the occurrence of serious side effects was equivalent between TNF-α and placebo receiving patients (RR = 0.83; 95%[0.69, 1.00], Z = 1.98, p = 0.05, Figure 8). Also, no significant difference was found between the anti-TNF-α group recipients and the cyclosporine recipients in terms of serious side effects (RR = 0.63; 95% [0.30, 1.34], Z = 1.19, p = 0.23, Figure 8).

## Discussion

Refractory UC treatment is one of the most challenging aspects in the clinical practice of luminal gastroenterology. UC patients who have frequent disease relapse, despite receiving the optimal conventional medical treatments, have few remaining non-surgical options. However, TNF-α inhibition offers a possible alternative therapy for UC patients who are treatment refractory or intolerant to corticosteroids and/or immunosuppressants. In the present study, we analyzed RCTs studying the efficacy of TNF-α blockers where the duration of patient follow-up continued for at least 12 weeks post initial treatment. We found that TNF-α blockers are effective and relatively safe therapies for maintaining long-term remission and preventing colectomy in patients with refractory UC. Of the available TNF-α blockers, infliximab and cyclosporine are comparable when used as rescue therapy in acute severe steroid-refractory UC.

UC is a chronic inflammation of the colon with states of disease that can range from dormant to refractory. Conventional therapy against UC includes a wide range of drugs, such as aminosalicylic acids, thiopurines, and corticosteroids. However, these agents fail to adequately control the disease in a large proportion of UC patients and are associated with many adverse side effects [34], [35]. It has now been recognized that treatment goals should go beyond just controlling the symptoms of UC. Rather, UC treatment should aim to rapidly induce steroid-free remission, and achieve complete mucosal healing, while minimizing serious complications and side effects [36]. Due to the introduction of newer biological therapies, such as anti-TNF-α, these treatment goals are within the realm of possibility.

Of the developed anti-TNF-α therapies, infliximab, adalimumab and golimumab have been approved by the Food and Drug Administration (FDA) for the treatment of UC. The efficacy of such agents in steroid-refractory UC was first shown in a controlled pilot study [23]. Later, however, a larger placebo controlled trial (n = 43) failed to support the efficacy of infliximab in active glucocorticoid resistant cases [11]. Subsequently, increasingly controlled trials were designed to assess the effect of infliximab and adalimumab on refractory UC. Two recent well controlled trials showed that golimumab could induce a clinical response, as evidenced by clinical remission and mucosal healing in patients with active UC [12], [13]. Unfortunately, both trials were excluded in our analyses due to a failure to follow-up with patients for at least 12 weeks after the initial treatment [12] and the enrolled patients are these who were response to golimumab therapy [13], respectively. Therefore, only infliximab and adalimumab were pooled for analysis within this study.

The rigorous inclusion criteria employed during our literature search returned 8 trials described in 7 published studies (n = 1922) that were hence pooled for meta-analysis. Among these studies, infliximab and adalimumab were compared to a placebo controlled group in 3[8], [28] and 1 trial(s) [9], respectively. The patients in the first 3 trials were randomized to receive infliximab at doses of 5 or 10 mg/kg via intravenous, or the matched placebo at weeks 0, 2, and 6, and then every 8 weeks[8], [28]. The patients in the fourth trial(s) were randomly assigned to receive subcutaneous injections of 160 mg adalimumab at week 0, 80 mg at week 2 and then 40 mg EOW beginning at week 4, or the matched placebo[9]. These studies concluded that anti-TNF-α therapy was slightly a little superior than administration of a placebo for treatment of UC patients in terms of clinical remission, mucosal healing, steroid-free remission, and reduction of colectomy rate, without causing serious side effects. Therefore, TNF-α blockers are an effective and relatively safe therapy to maintain long-term remission and avoid colectomy for patients who are not responsive to conventional treatment. Additionally, 3 small trials (n = 57) compared infliximab to steroid treatment. There were no statistically significant difference found in terms of frequency of clinical remission, mucosal healing and colectomies. However, this conclusion is unreliable due to the low number of patients in these trials. Moreover, one RCT trial (n = 115) compared infliximab to cyclosporine for use as rescue therapy for acute severe UC patients who were not responsive to intravenous steroid treatment. It was found that these drugs were comparable for rate of clinical remission, mucosal healing, colectomies rate and serious side effects. This result confirmed the conclusions from a previous meta-analysis, which pooled six retrospective cohort studies but did not include RCTs [37].

Besides the efficacy, the possible side effects of TNF-α blocker treatment were of interest when conducting this study. The main side effects that have been recorded are an increased risk of infections, occurrence of autoimmune disorders [28], and risk of lymphoma or other malignancy [9]. In the present study, we found the risk of serious side effects were similar between anti-TNF-α and the control (p<0.00001, Figure 8), Overall, serious side effects occurred in 20% of patients within the placebo group and 16.9% of patients within the anti-TNF-α group. However, the rate of adverse events (AE) for AE's for combined immunomodulator/anti-TNF therapy compared to each used as monotherapy beyond conventional treatment is a source of controversy. More studies with larger sample size are needed in future trials to further evaluate the rate serious infection due to the limit sample size in the current ones.

When performing a meta-analysis, caution needs to be used when drawing conclusions based on pooled studies of heterogeneous patient populations. To control for this heterogeneity, only the studies that had enrolled patients refractory to conventional treatment (e.g. steroid-dependent, nonresponsive to intravenous steroid or nonresponsive to conventional therapy) were included. Furthermore, trials of only a single infusion of anti-TNF-α and/or and a patient follow up duration of less than 12 weeks were excluded. To statistically control any further heterogeneity in the meta-analysis, we used a random effects model to analyze if there was heterogeneity among the trials. Also, subgroup analyses were performed based on the interventions applied in the control group. It should be noted that the majority of the included studies were judged to be of “moderate to high” quality without publication bias during our analysis.

Despite rigorous inclusion criteria that have been made to reduce the heterogeneity there are still several limitations within this study. First, the duration of patient follow up within the analyzed trials was still variable, ranging from 13 weeks to 54 weeks. Second, UC severity was not uniform upon trial initiation. Some trials enrolled patients that were steroid-dependent/refractory, while others enrolled those nonresponsive to intravenous steroid therapy and/or oral conventional drugs treatment. Third, the co-therapy scheme and dose administered of TNF-α blockers differed between trials. All of these instances of variability could affect the results drawn from our analysis.

In summary, this meta-analysis has updated the UC treatment field and demonstrated that TNF-α blockers were superior for patient treatment as compared to placebo. This conclusion was based on increased achievement of clinical remission and mucosal healing and reduction in the need for colectomy, combined with no significant, severe side effects. Using anti-TNF-α also spares patients the effects of corticosteroid treatment, which is used when the patients have refractory UC nonresponsive to conventional treatment. Additionally, infliximab and cyclosporine are comparable when used as rescue therapy in acute severe steroid-refractory UC, although, more randomized trials are needed to further evaluate the efficacy of these agents. So, in selected patients with moderate to severe active ulcerative colitis who have failed to respond or are poorly responsive to standard pharmacologic forms of treatment with corticosteroids and immunosuppressive agents, therapy with an anti-TNF-α agent may be considered. In addition, it may be necessary to identify biomarkers that indicative of patients who will respond to the TNF-α inhibitor.

## Supporting Information

Checklist S1 **PRISMA checklist.**
(DOC)
